# Natural Changes in Brain Temperature Underlie Variations in Song Tempo during a Mating Behavior

**DOI:** 10.1371/journal.pone.0047856

**Published:** 2012-10-24

**Authors:** Dmitriy Aronov, Michale S. Fee

**Affiliations:** Department of Brain and Cognitive Sciences, McGovern Institute for Brain Research, Massachusetts Institute of Technology, Cambridge, Massachusetts, United States of America; Claremont Colleges, United States of America

## Abstract

The song of a male zebra finch is a stereotyped motor sequence whose tempo varies with social context – whether or not the song is directed at a female bird – as well as with the time of day. The neural mechanisms underlying these changes in tempo are unknown. Here we show that brain temperature recorded in freely behaving male finches exhibits a global increase in response to the presentation of a female bird. This increase strongly correlates with, and largely explains, the faster tempo of songs directed at a female compared to songs produced in social isolation. Furthermore, we find that the observed diurnal variations in song tempo are also explained by natural variations in brain temperature. Our findings suggest that brain temperature is an important variable that can influence the dynamics of activity in neural circuits, as well as the temporal features of behaviors that some of these circuits generate.

## Introduction

Many behaviors, such as displays of emotion, aggression and courtship rituals, as well as communication signals, are produced differently depending on the social context in which the behavior is generated [1], [2]. Likewise, the song of an adult male zebra finch varies slightly with social and behavioral context. Some songs are directed toward a nearby female bird (“directed” singing); other songs, like those produced in social isolation, are not directed toward a female (“undirected” singing) [3]. Though the two song types consist of the same repeated sequence of sounds (“motif”), directed song is produced in a more stereotyped fashion than undirected song [3]–[8]. Furthermore, directed singing is also delivered with a faster tempo than undirected singing [3], [5], [8]–[11].

Much work has been devoted to the mechanisms underlying the difference in vocal variability between directed and undirected songs [4]–[8]. Variability is thought to depend on neural activity in a premotor nucleus LMAN (lateral magnocellular nucleus of the nidopallium) – a cortical output region of an avian basal ganglia thalamocortical circuit [12]. Activity of LMAN neurons is highly variable across song trials and changes with social context [4], [6], [13], [14]. Furthermore, bilateral lesions and inactivations of LMAN reduce song variability [5], [8], [13]–[15], eliminating the difference in variability between directed and undirected songs [5], [8]. These and other studies have led to the suggestion that changes in LMAN activity are responsible for the social modulation of song variability, and that neuromodulatory inputs into LMAN or associated basal ganglia circuits may mediate these changes [7], [9], [16]–[19].

Similarly, studies have hypothesized that LMAN activity is also responsible for context-dependent changes in song tempo [5], [9], [10]. Although some previous work lends support to this hypothesis [5], [20], a recent study showed that inactivations of LMAN do not eliminate the difference in tempo between directed and undirected singing [8]. Thus, unlike changes in song variability, changes in tempo may be driven by a different mechanism that is independent of LMAN activity. In addition, singing also exhibits a diurnal variation in tempo of a magnitude similar to that produced by social context [11]. The mechanistic origins of these slow variations in song tempo have also not been identified.

Song production depends critically on activity of another nucleus, HVC (used as a proper name) [21], [22], which, like LMAN, is located in the avian cortex [23]. Neurons in HVC exhibit brief bursts of spikes, each occurring at a different time in the song, leading to the hypothesis that HVC codes for song timing [24], [25]. Recent work has shown that mildly cooling HVC (by several degrees below normal brain temperature) uniformly slows down song features on all timescales, by about 3% per °C [26]–[28]. Yet cooling the downstream premotor area RA (robust nucleus of the arcopallium) does not change song timing. Because cooling slows down all biophysical processes, these results suggest that the circuit dynamics within HVC actively generate song timing, perhaps via the propagation of activity through a synaptically-connected neuronal chain [29]–[32].

It has been shown that brain temperature can rise naturally during increased activity or in the presence of salient stimuli like food or other animals [33]–[36], leading us to wonder if the faster song tempo during directed singing results from an increased brain temperature in the male zebra finch, triggered by the presence of the female. It is also known that brain temperature in warm-blooded animals can exhibit diurnal variations of up to 1-2°C [33], [37], suggesting that slow variations in song tempo throughout the day may also be related to changes in brain temperature. To test these hypotheses, we recorded brain temperature in freely behaving male zebra finches and examined the temperature response to the presentation of a female bird, as well as the relation to the time of day. We observed natural variations in brain temperature that were strongly correlated with both social context and with the time of day. These temperature changes fully account for the observed social and diurnal modulations of song tempo.

## Results

**Figure 1:**
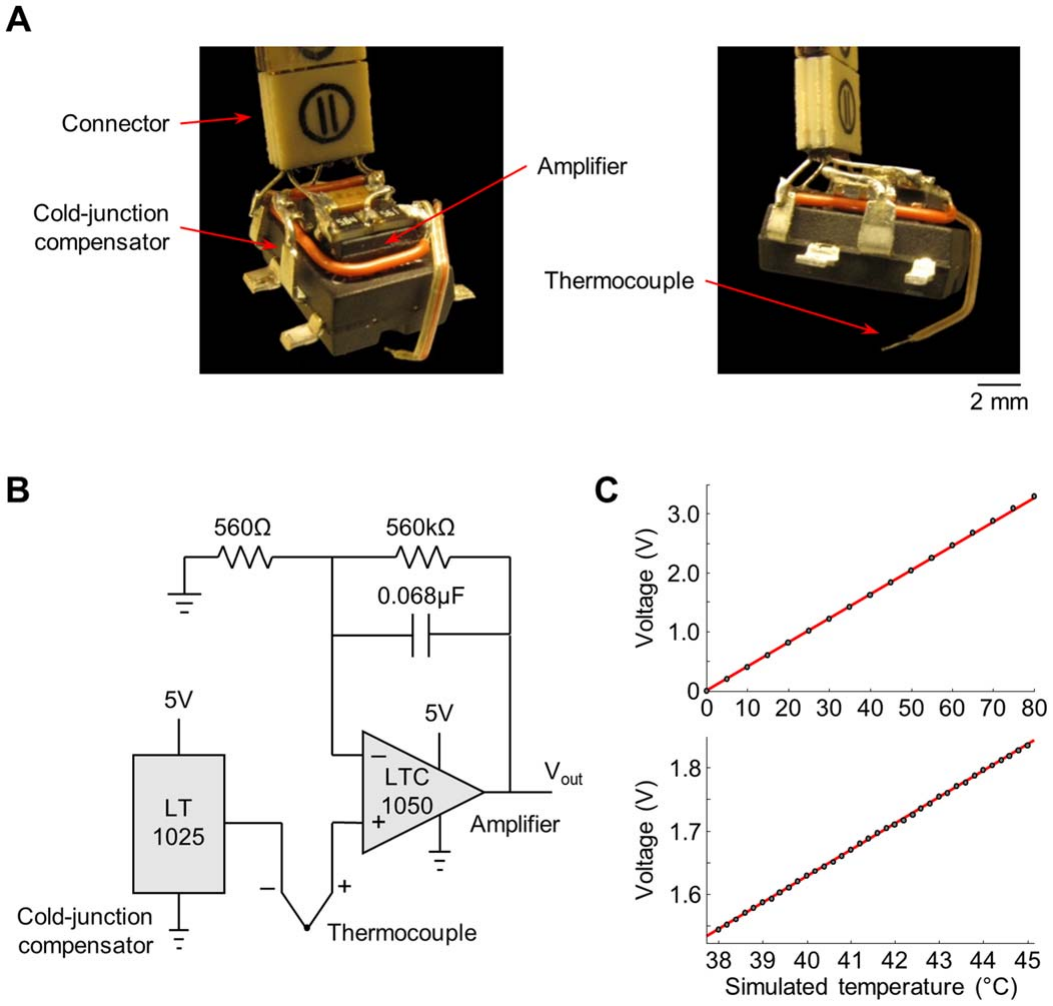
Method for recording brain temperature in freely behaving zebra finches. (A) Photographs of the miniature device for brain temperature recordings. (B) Circuit diagram of the device. (C) Voltage output (V_out_) produced by the device at a wide range of simulated temperature values (top) and at values in the physiological range (bottom). Simulated temperatures were produced by a commercial thermocouple calibrator. Symbols indicate averages of 10-s recordings; 95% confidence intervals are smaller than the symbols. Red lines are the linear fits to the data.

**Figure 2:**
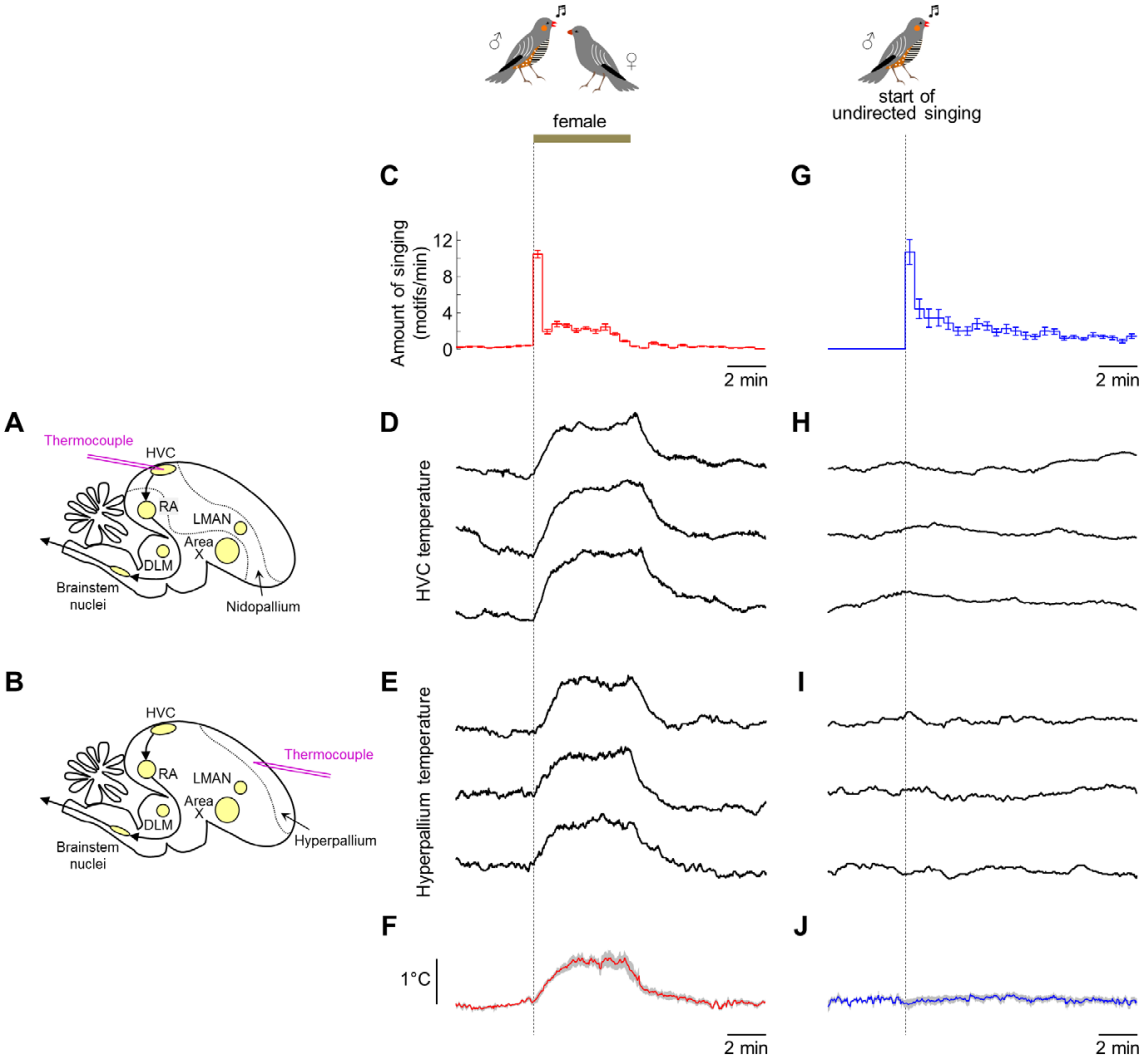
Brain temperature in male zebra finches rises in response to presentation of a female bird. (A, B) Schematic diagrams of the implantation of a thermocouple for temperature measurements. (A) A thermocouple was implanted into HVC along the approach angle shown. Yellow ovals indicate major nuclei of the song system, and arrows indicate the motor pathway. (B) In other experiments, a thermocouple was implanted into the hyperpallium, outside of the song system. Dotted line indicates boundary of the hyperpallium. (C) Average amount of singing triggered by presenting a female bird to the male. Error bars are SEM across all birds. (D) Examples of temperature recordings in HVC of bird #3 during individual trials in which a female bird was presented to the male for 5 min. (E) Examples of temperature recordings in the hyperpallium of bird #5 during presentations of a female. (F) Average temperature change across all 8 male birds during presentations of a female. Shaded area is SEM across all birds. (G) Average amount of singing produced in social isolation aligned to the onsets of the epochs of undirected singing. Epochs were defined as singing periods preceded by at least 20 min without singing. (H) Examples of HVC temperature recordings from the same bird as in (D) during epochs of undirected singing. (I) Examples of hyperpallium temperature recordings from the same bird as in (E) during epochs of undirected singing. (J) Average temperature change in all 7 birds during epochs of undirected singing. For all averages, data were first averaged for each bird; mean values were then averaged across all 8 birds.

To record brain temperature in freely behaving birds, we designed and built a miniature temperature transducer incorporating a small thermocouple (Figure 1; see Methods). Brain temperature was measured in HVC of 5 freely behaving male birds (Figure 2A). In 3 additional birds, we recorded temperature at the same depth as HVC, but in a region of the hyperpallium – a cortical area outside of the song system (Figure 2B; see Methods). Female birds were presented to the subjects for 5-min periods, separated by at least 20 min of isolation. On all trials recorded, birds sang in response to the presentation of the female, typically producing a long bout of singing immediately upon presentation, followed by several additional bouts throughout the remainder of the 5-min period (Figure 2C). In all birds, HVC temperature increased in response to the presentation (Figure 2D), reaching an average value of 0.94±0.19°C above baseline after 2 min (p<0.001; ±SEM and t-test here and elsewhere, unless stated otherwise). In the hyperpallium, temperature changes triggered by the presentation of the female were similar to those measured in HVC (Figure 2E; 0.89±0.20°C), suggesting that the observed temperature change was global in the brain; we subsequently pooled data from these two groups of birds for analysis (Figure 2F; N = 8 birds).

We wondered if these temperature changes were specifically related to the presentation of the female bird or if they resulted, indirectly, from the elicited singing. To address this question, we analyzed temperature measurements at onsets of epochs of undirected singing (in social isolation) that were preceded by at least 20 min without singing (Figure 2G–J). Temperature did not increase following the onset of undirected singing (0.049±0.072°C change, p = 0.53, N = 7 birds), even though the amount of singing during these epochs was similar to the amount of singing produced upon the presentation of the female (Figure 2C and G; p = 0.68 in the first 2 min). Thus, temperature changes appear to be triggered by the presentation of the female, rather than by the act of singing itself.

**Figure 3:**
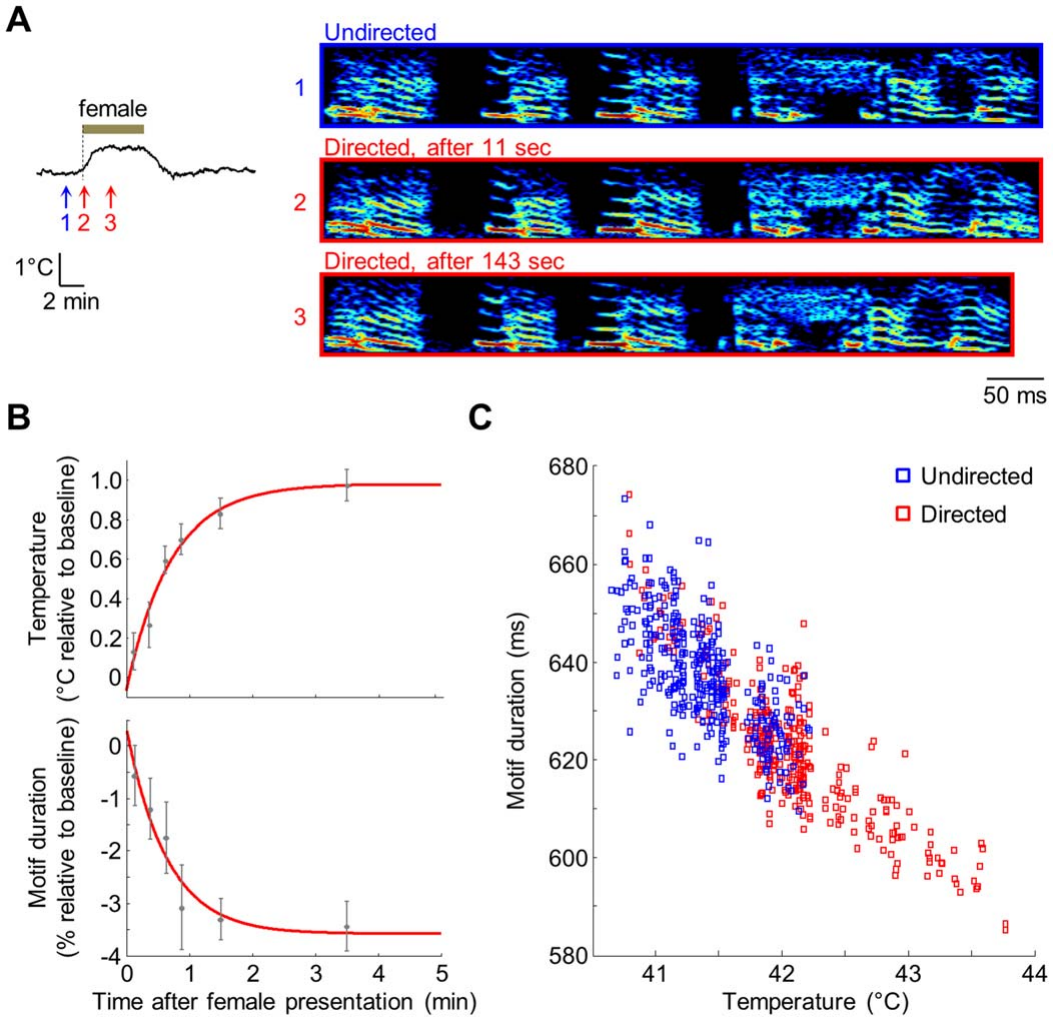
Song tempo is strongly correlated to brain temperature. (A) Left: Temperature recording in HVC of bird #2 during a presentation of a female. Right: Spectrograms from the same trial of an undirected motif (at time point 1 indicated on the temperature recording) and two directed motifs – one produced almost immediately after the presentation and another produced >2 min later. Note that the directed motif produced several minutes after the presentation (time point 3) has a faster tempo than the undirected motif (time point 1), while the motif produced immediately after the presentation (time point 2) has a slow tempo similar to the undirected motif. (B) Time-dependent changes of brain temperature (top) and motif duration (bottom) following presentation of a female. Data points are averages in four 15-s bins from 0-1 min, one bin from 1-2 min, and one bin from 2-5 min. Data were first averaged across presentations for each bird; mean values were then averaged across all 8 birds. Error bars are SEM across all birds. Red traces are exponential fits to the data. Baseline duration for each bird is the average duration of undirected motifs; baseline temperature is the average temperature during these motifs. (C) Temperature and motif duration for all motifs produced by the same bird as in (A).

We next examined the timecourse of changes in brain temperature and changes in song tempo. Because birds produced directed singing immediately upon the presentation of the female bird, while brain temperature changed on a slower timescale, we could disambiguate the role of social context from that of brain temperature. We found that changes in song tempo, quantified by measuring the duration of the song motif [11], did not occur immediately upon the presentation of the female (Figure 3A), but followed a slow timecourse nearly identical to the timecourse of changes in brain temperature (Figure 3B; time constants: 40.8±4.6 s for temperature, 43.8±9.6 s for motif duration, p = 0.70, N = 8 birds). Indeed, directed motifs sung immediately after the presentation of the female – when the male appeared to be most intensely directing his attention to the female – had the same duration as baseline undirected motifs (p = 0.78 for the exponential fit at 0 s).

In all recorded birds, variations of directed motif durations were strongly correlated with variations in brain temperature (Figures 3C and S1; p<0.001, r^2^ = 0.56±0.05, N = 8 birds). The linear relationship between fractional change in motif duration and temperature had a slope of -3.23±0.25% per °C (see Methods), similar to that observed by direct manipulation of HVC temperature [26]. Durations of undirected motifs were also strongly correlated with temperature (p<0.01 in all birds, r^2^ = 0.23±0.08, N = 7 birds). However, as expected, the lower r^2^ value suggests that undirected singing exhibited more variability unrelated to temperature than did directed singing. To determine whether temperature accounted for the difference in tempo between directed and undirected songs, we subtracted the linear fit to temperature, calculated on only directed motifs, from all motif durations (see Methods). Following this subtraction, the difference between average directed and undirected motif durations was reduced by 90.5±8.8%. Thus, most of the context-dependent difference in tempo could indeed be explained by a linear dependence on temperature.

**Figure 4:**
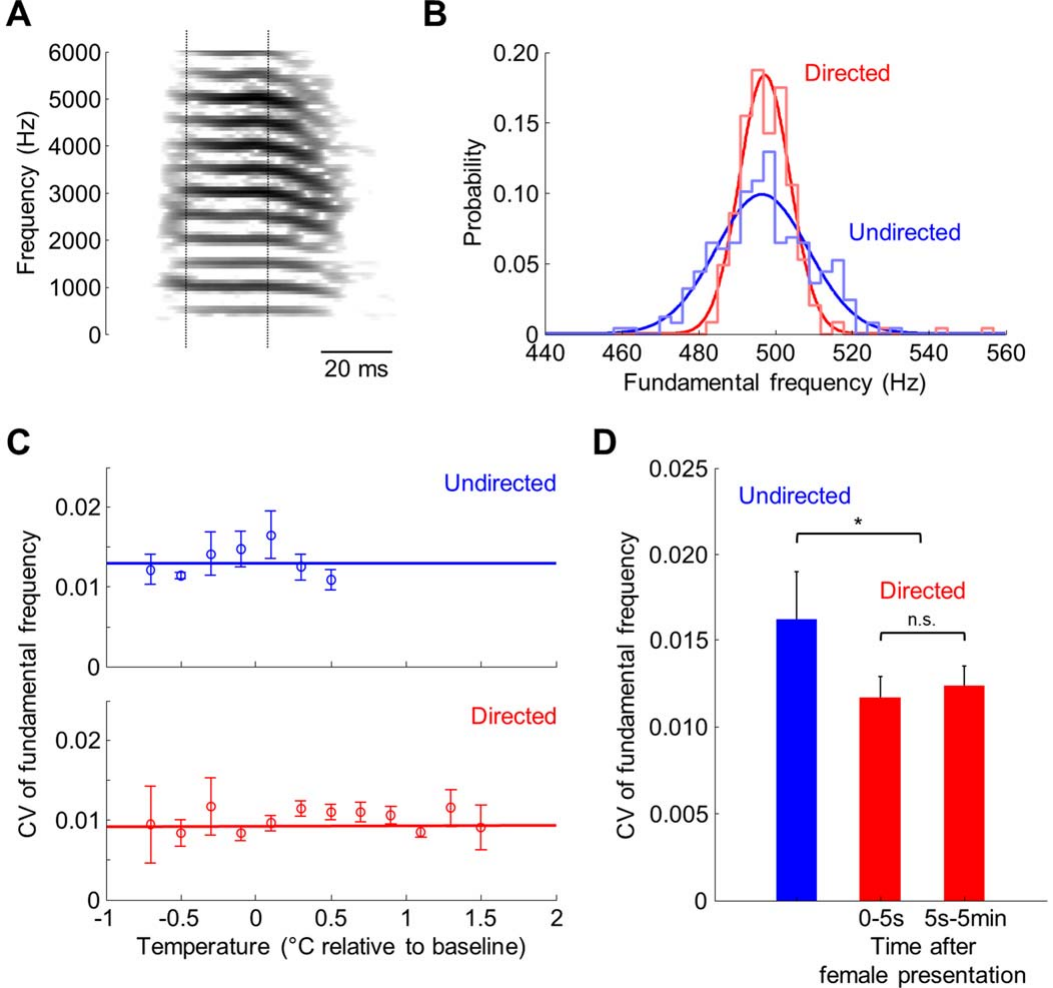
Acoustic variability is not correlated to brain temperature. (A) Spectrogram of a syllable from bird #7 that contained a harmonic stack and was used for the calculation of fundamental frequency. Dotted lines indicate the automatically detected time interval on which fundamental frequency was calculated. (B) Distributions of fundamental frequency across all directed (red) and undirected (blue) renditions of the harmonic stack shown in (A). Gaussian fits to both distributions are also shown. Note that harmonic stacks during directed singing exhibit less variability. (C) Variability of fundamental frequency measured across different temperature values. All harmonic stacks were sorted into temperature bins (0.2°C wide); the coefficient of variation of the fundamental frequency (CV, standard deviation/mean) was measured within each bin. Lines are linear fits to the data. Baseline temperature is the average temperature recorded during all undirected motifs. (D) CV of the fundamental frequency for all undirected (blue) and directed (red) songs. For directed song, harmonic stacks are separated into those produced within 5 s after the presentation of a female and those produced after 5 s. Note that the variability is already reduced in songs produced immediately upon presentation. In (C) and (D), data shown are average CV values across harmonic stacks from 8 different syllables in 5 birds for directed songs and 7 syllables in 4 birds for undirected songs; error bars are SEM across harmonic stacks.

In addition to exhibiting a faster tempo, directed singing is known to be less variable than undirected singing [3]–[8]. We wondered whether this difference between the two song types had any dependence on brain temperature. To quantify the variability of singing, we focused on one commonly measured acoustic feature – the fundamental frequency of those sounds within the song that have clean harmonic structure (“harmonic stacks”) [5], [8], [13]. We identified 8 harmonic stacks in 5 of the recorded birds (Figure 4A; 3 birds with 1 stack, 1 bird with 2 stacks and 1 bird with 3 stacks each, see Methods) and quantified variability of each stack by measuring the coefficient of variation (CV) of its fundamental frequency across all recorded syllables. As expected, most harmonic stacks exhibited greater variability during undirected than during directed song (e.g. Figure 4B; p<0.01 for 6 stacks in 3 birds out of the 7 stacks in 4 birds that were recorded during both song types, bootstrap statistical test carried out separately on each harmonic stack).

To analyze the dependence of acoustic variability on brain temperature, we sorted harmonic stacks into bins of temperature values at which they were produced and quantified the CV of fundamental frequency in each temperature bin. For both undirected and directed songs, the average CV across harmonic stacks was not correlated to temperature (Figure 4C; undirected: p = 0.99, N = 7 stacks in 4 birds; directed: p = 0.16, N = 8 stacks in 5 birds). Furthermore, acoustic variability appeared to decrease immediately following the presentation of the female: harmonic stacks produced in the first 5 s of directed singing after the presentation had a lower CV than those stacks produced during undirected singing (Figure 4D; p<0.05 in 5 of 7 stacks from 4 of 5 birds). Also, harmonic stacks produced in the first 5 s of the presentation had the same CV as those stacks produced after 5 s (p = 0.45, N = 8 harmonic stacks in 5 birds). These results illustrate that the dependence on temperature is specific to song tempo: whereas song tempo is correlated to brain temperature and follows a slow timecourse after the presentation of the female, acoustic variability is reduced immediately in response to the female and shows no dependence on temperature.

**Figure 5:**
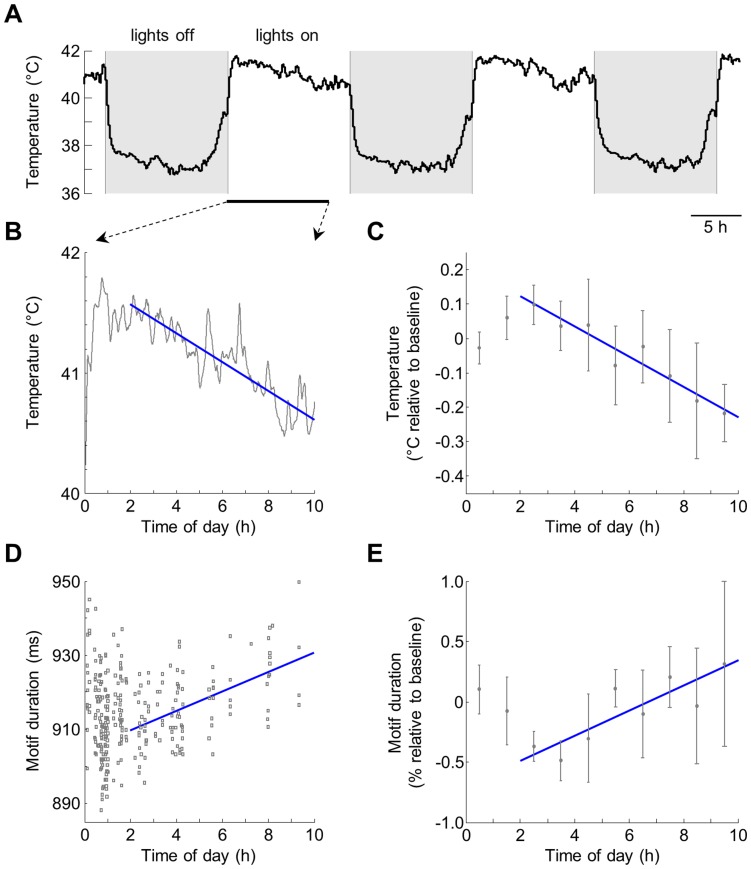
Brain temperature explains circadian fluctuations in song tempo. (A) Continuous brain temperature recording in bird #5 during >2 continuous days of social isolation, showing large changes between sleeping a waking hours. (B) Detail showing temperature during waking hours 0–10 of a single recording day. (C) Temperature across all birds (N = 6), averaged in 1-h bins. (D) Durations of all motifs produced by the bird during hours 0–10 of the same recording day as in (B). Each symbol is an individual undirected motif. (E) Motif duration across all birds, averaged in 1-h bins. In all panels, blue lines are linear fits to data from 2–10 h. Baseline values are averages across the first 2 hours of the day, and error bars are SEM across birds. For averages, values were first averaged across days; mean values were then averaged across birds.

In addition to the context-dependent changes in tempo, it has been reported that songs exhibit consistent slow changes in tempo throughout a day of singing [11]. We asked whether these variations can also be explained by changes in brain temperature. In 6 birds, we recorded singing and brain temperature continuously through several days, during which the bird was socially isolated. In addition to large differences between sleeping and waking hours (Figure 5A), brain temperature also exhibited consistent daytime changes (Figure 5B and C). There was a peak in brain temperature roughly 2 h after lights-on, followed by a gradual decline throughout the rest of the day (-0.032±0.007°C/h during hours 2–10, p<0.001, N = 6 birds). Motif duration showed the opposite pattern, exhibiting a minimum at roughly 2 h after lights-on, followed by a slow increase during hours 2–10 (Figure 5D and E; 0.096±0.036%/h, p<0.01); this pattern is similar to the previously described trend [11]. During these slow diurnal variations, the slope of the relation between fractional changes in motif duration and brain temperature was -2.77±0.51%/°C, again similar to that observed by direct manipulation of HVC temperature [26]. To determine whether temperature accounted for diurnal tempo variations, we subtracted the linear dependence on temperature from all motif durations. After this subtraction, no residual dependence on time of day was detectable (slope 0.004±0.029%/h during hours 2–10, p = 0.24, see Methods), suggesting that slow diurnal variations in song tempo are fully explained by slow variations in brain temperature.

## Discussion

We observed changes in brain temperature of zebra finches related both to the social context and to the diurnal cycle. In both cases, brain temperature was strongly correlated to song tempo, and the slope of this relationship was approximately −3%/°C. This correlation likely reflects a causal effect of temperature on song tempo, since direct manipulations of HVC temperature have been shown to induce changes in tempo of a similar magnitude (-3%/°C) [26], [28]. Changes in brain temperature explained nearly all of the variance in song tempo related to social context and to the time of day, suggesting that these natural changes are a predominant mechanistic contributor to both of these sources of variation in song tempo.

Notably, temperature in HVC increased only during directed, but not during undirected singing, even though neural activity in this premotor region is expected to be largely the same in these two conditions. This result suggests that the temperature increase is not likely to be caused by local activity-dependent changes within HVC, such as an increase in the metabolic rate of HVC neurons or a local change in blood flow, but may be more widespread in the brain. In support of this hypothesis, we observed increases in temperature even in the hyperpallium, a cortical area outside of the song system. Such widespread temperature changes would be consistent with studies in other warm-blooded species that found global temperature variations, of a similar magnitude to what we observe, during a variety of motivated behaviors and in response to salient stimuli [36].

One consideration is that the hyperpallial location we recorded from is part of the visual system [38]. Some of the temperature change measured in this area during directed singing could, in principle, have resulted from local metabolic activity induced by the visual presentation of the female bird. However, since we found no evidence of local activity-dependent temperature changes in HVC, it is unlikely that temperature changes in the hyperpallium are due to local visual stimulus-induced neural activity.

Our results raise a question of which specific aspects of the behavioral response could induce global changes in brain temperature. In other species, temperature changes similar to those we report here have been shown to depend on a number of diverse factors, including sensory stimulation [36], sexual arousal [34] and movement [33], [35]. Each of these factors changes in male birds during the presentation of a female and directed singing [39]. In addition, male zebra finches exhibit an increase in heart rate during presentations of female birds [10], and changes in blood circulation have also been shown to cause variations in brain temperature [36]. Thus, an increase in temperature is likely to be caused by a combination of physiological factors, and further studies are necessary to characterize their relative contributions.

Previous studies have shown that LMAN activity drives changes in acoustic variability between directed and undirected songs [5], [8], [13], and some have suggested that a similar LMAN-dependent mechanism is responsible for the difference in song tempo [5], [9], [10]. However, other studies have shown that pharmacological inactivation of LMAN [8], or neuromodulatory manipulation of the basal ganglia-forebrain pathway that includes LMAN [19], affect song variability but not tempo. Our analysis of the timecourse of behavioral changes provides further evidence that a mechanism outside LMAN drives social context-related changes in song tempo. First, we found that changes in acoustic variability, which depends on LMAN activity, were fast, appearing within the first five seconds after the presentation of the female. Consistent with this, changes in LMAN activity from undirected to directed singing are also fast and are observed during song motifs that immediately follow the presentation [4], [6]. Second, we found that, in contrast to the rapid timecourse of changes in variability, changes in song tempo were slow, taking close to two minutes to reach equilibrium. This slow change precisely matched the timecourse of brain temperature changes. Our results therefore suggest that two distinct mechanisms underlie changes in song across social contexts: changes in variability are driven by fast neural mechanisms involving LMAN, while changes in tempo are independently driven by slow variations in brain temperature.

Although temperature explained >90% of the difference in tempo between directed and undirected songs, our results leave open the possibility that small residual differences may be influenced by other mechanisms, such as LMAN activity. Inactivating LMAN, while measuring temperature and accounting for the time elapsed after the presentation of a female, may be necessary to address this possibility.

Our findings suggest that estimates of the social context-related changes in tempo will depend strongly on how directed song is elicited experimentally and on which songs are analyzed. Previous studies of the difference between directed and undirected songs vary widely these criteria and also vary widely in the magnitude of the tempo change reported. Typically, all directed songs are considered, including those produced immediately after the presentation of the female [5], [8]. Because temperature changes gradually after the presentation, such analysis will include the slower directed motifs produced initially, and will therefore underestimate the effect of social context on tempo. Indeed, these studies reported differences in tempo smaller than those we observed when comparing songs at steady-state temperature values. Other studies include directed songs produced after the male and the female have been kept together for a long period of time [10], [11]. These studies also reported smaller differences than those we observed, perhaps because habituation of the male to the female may have reduced brain temperature. In fact, habituation of temperature responses to salient stimuli has been observed in other species [36].

Also, because brain temperature changes gradually, estimates of average song tempo will depend strongly on the distribution of latencies of song bouts after the presentation of the female. The distribution of latencies can, in turn, depend on many factors, such as subtle changes in arousal, recovery from surgery, and the amount of time elapsed from previous presentations of female birds. These factors can influence the interpretation of some experimental results, such as the report that LMAN lesions eliminate the difference in tempo between undirected and directed songs [5]. A possible explanation of this result is that, following lesions, birds sang directed songs with a shorter average latency. For instance, these birds may have still produced a large initial song bout in response to the female (e.g. Figure 2C), but sang less during the rest of the presentation period due to nonspecific effects of the surgical manipulation. Thus, when averaged across the entire presentation period, the estimated difference in tempo between directed and undirected songs would be largely eliminated, even though the LMAN lesion had no actual effect on the magnitude of the tempo change. In contrast, this hypothetical effect would be unlikely to occur during a less behaviorally disruptive method of LMAN silencing. Indeed, no effect on tempo was observed during pharmacological inactivation of LMAN using reverse microdialysis [8].

Temperature changes are known to influence simple behaviors in invertebrates and cold-blooded vertebrates that are driven by central pattern generators (CPGs), such as rhythmic movements and sound production [40]–[45]. Because these animals lack internal thermal homeostasis, even natural ambient temperature fluctuations can affect the tempo of their behaviors [41]. Our results show that, in spite of efficient homeothermic mechanisms, internal brain temperature fluctuations can produce observable changes in behavior even in a warm-blooded animal.

Mammals have been shown to exhibit changes in brain temperature across behavioral conditions similar in magnitude to those we report here [33]–[36]. Our results therefore raise an interesting possibility that brain temperature is an important dynamical variable in the functioning of other neural circuits that generate behaviors, including those in mammals. Especially in the context of changing states of arousal, one is left to wonder how much of the variance in observable timescales – such as psychophysical reaction times and frequencies of intrinsic neural oscillations – can be explained by natural variations in brain temperature.

## Methods

### Animal Subjects

Subjects were adult zebra finches (>100 days-post-hatch) obtained from the Massachusetts Institute of Technology breeding facility. Male birds that produced consistently large amounts of directed singing in response to the presentation of a female bird were selected for experiments. Animal care and experiments were carried out in accordance with the National Institute of Health guidelines and approved by the MIT Institutional Animal Care and Use Committee.

### Sound Recordings

During the experiments, a single male bird was kept in a small cage inside a sound isolation chamber and maintained on a 12h:12h day-night light cycle. Sound was recorded with a G.R.A.S Sound and Vibration 40AE microphone and digitized at 40 kHz. Recordings on a computer were done with Sound Analysis Pro [46] or custom Matlab software, which both detected songs and triggered recording only when singing occurred. To elicit directed singing, a small cage with an adult female bird was quickly positioned within ∼15 cm of the male bird’s cage, with minimal disturbance of the male before the female’s cage was in place. The female was presented for a total of 5 min and was then immediately removed. Successive presentations were separated by periods of at least 20 min. For recordings of diurnal changes in song tempo and brain temperature, subjects were socially isolated and otherwise undisturbed for a continuous period of 2–5 days and were monitored by video camera.

### Brain Temperature Measurements

Brain temperature was recorded in freely behaving birds using a thermocouple (40-gauge type-K thermocouple, Omega) and a custom designed and built miniature temperature transducer (Figure 1A). The thermocouple was connected to a cold-junction compensator (Linear Technology LT1025ACN8) that generated a voltage offset, which was added to the thermocouple signal (Figure 1B). This offset compensated for nonlinearities in the voltage-temperature relationship of the thermocouple and ensured an output of 0 V at 0°C, regardless of the internal temperature of the device itself. The output was amplified and low-pass filtered at ∼4 Hz using a precision low-drift operational amplifier (Linear Technology LTC1050CS8). A miniature connector (Omnetics) and a thin, flexible cable were used to read signals and to deliver power to the electronics. For recordings in behaving birds, the cable was attached to a custom torque-sensitive commutator system [47]. Readings from the device were digitized together with sound (at 40 kHz) and recorded on computer only when singing was detected. In addition, uninterrupted temperature recordings were obtained at 100 Hz sampling during presentations of female birds and at 1 Hz sampling continuously throughout the day for the analysis of diurnal patterns.

We used a thermocouple calibrator (Omega CL-3512A) to simulate temperature inputs for testing and calibrating our devices. As expected from the design, device output (after amplification) was linear with a slope of 41.0 mV/°C across a wide range of simulated temperatures, including values in the physiological range (Figure 1C). This value was used to convert voltage readings from the device to temperature.

In experimental birds, temperature was recorded either in the center of HVC (N = 5 birds) or in an area outside of the song system (N = 3 birds). For the latter group of birds, we chose a location in the hyperpallium [23], 5.5 mm anterior to HVC. Previous work with immediate early gene expression indicates that this location is likely to be within a region of the forebrain visual system [38]. The location was chosen to be at the same depth as the center of HVC (500 µm) in order to avoid possible differences arising from dorso-ventral temperature gradients in the brain [36].

### Surgery

For device implantation, birds were anesthetized with 1–2% isofluorane in oxygen and placed into a stereotaxic apparatus. A small craniotomy and duratomy were made either above HVC or above the targeted location in the hyperpallium (see above). The tip of the thermocouple was then lowered into the center of HVC or into a location at an equivalent depth (500 µm). In both cases, the thermocouple was inserted as parallel to brain surface as possible (<30°) in order to minimize possible heat transfer along the thermocouple. The device was attached to the cranium with light-cured dental acrylic (Pentron Clinical). Birds typically began to sing the day after the surgery; temperature measurements were started 2–4 days after the surgery.

### Measurement of Motif Durations

We used a method similar to that developed by Glaze and Troyer [11] for precisely extracting motifs from song recordings. To detect motif onsets, sounds were band-pass filtered between frequencies 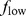 and 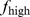 with an order-200 FIR filter (Matlab functions fir1 and filtfilt). The choice of 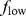 and 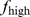 is discussed below. Sound amplitude was then calculated by squaring the filtered signal, smoothing with a 2.5-ms square window and computing the logarithm. We then approximated the derivative of the amplitude signal by computing the difference between adjacent amplitude values. The derivative was further smoothed with a 2.5-ms square window. Pass-band cutoff frequencies 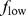 and 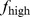 were manually chosen for each bird such that each motif onset corresponded to a sharp and easily identifiable positive peak in the amplitude derivative; the locations of these peaks were detected manually and defined as motif onsets. Across birds, chosen 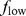 values were between 500 and 3000 Hz, and 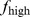 values were between 1500 and 8000 Hz. The same procedure was repeated to detect motif offsets, generally with a different choice of 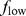 and 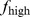 in the same range. Motif offsets were defined by locations of negative peaks in the amplitude derivative signal.

### Analysis of the Relationship between Motif Duration and Brain Temperature

For each song motif, we assigned a single temperature value, defined as the average temperature during that motif. In each bird, motif duration and temperature were strongly correlated and exhibited a linear relationship (Figures 3C and S1; see Results). To measure the slope of this relationship in normalized units, we defined baseline duration for each bird as the average duration of all undirected motifs. In one bird, no undirected motifs were recorded; for this bird, the baseline was defined as the 75th percentile of directed motif durations, which roughly corresponded to undirected motif durations in all other birds. Motif durations were then expressed in units of % above or below the baseline; dependence on temperature was expressed in units of %/°C and could be directly compared to results from direct temperature manipulations [26].

We used the following procedure to estimate how much of the difference between directed and undirected motif durations could be explained by temperature changes. For each bird, we first calculated the difference between the average undirected motif duration, 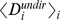 and the average duration of those directed motifs that were produced 2–5 min after the presentation of the female (once temperature had stabilized), 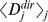. Here, 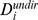 and 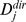 are the durations of the *i*th undirected motif and the *j*th directed motif (including only those at 2–5 min), respectively. The brackets 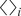 denote an average across all motif indexed by *i*. The difference was therefore 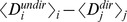. We then calculated a linear fit 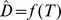 to the motif duration-temperature relationship for all directed motifs; here *T* is temperature, 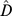 is estimated duration, and *f* is a linear function. This fit was then subtracted from all data points (directed and undirected motifs), and the difference between durations was again compared. To estimate how much of the difference between directed and undirected motifs was explained by temperature, we therefore used the following equation:
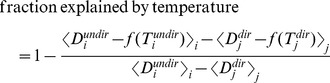
where 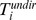 and 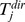 are temperature values recorded during undirected and directed motifs, respectively.

To analyze the dependence of motif duration and brain temperature on the time of day, brain temperature and motifs (undirected song) were recorded continuously through several days, during which the bird was maintained in social isolation. Both temperature and motif duration exhibited consistent slow changes following about 2 h after lights-on (see Results). We therefore analyzed the dependence on the time of day by fitting a line to brain temperature between 2 and 10 h after lights-on (Figure 5B and C; hours 10–12 were not included in analysis because none or very little singing was produced during this period). A line was also fit to motif durations measured over this period (Figure 5D and E). To determine whether the dependence of motif duration on the time of day could be explained by temperature, we calculated a linear fit to the motif duration-temperature relationship for all undirected motifs produced by the bird. This linear fit was then subtracted from all motif durations and the dependence on time of day was reevaluated between 2 and 10 h after lights-on.

### Analysis of Acoustic Variability

To quantify the acoustic variability of singing, we measured variations in the fundamental frequency of harmonic stacks across song renditions [5], [8], [13]. Harmonic stacks are sounds present in the songs of many zebra finches that exhibit clear harmonic structure and that maintain a stable frequency throughout a prolonged period of time (tens of milliseconds). These sounds allow accurately measuring fundamental frequency and comparing a single frequency value across song renditions.

We automatically identified harmonic stacks in the songs of each bird using a cepstrum-based pitch calculation method [48]. For each motif, we calculated the cepstrum (Fourier transform of the spectrogram). The cepstrum is a matrix that indicates harmonic power of sound across time (columns) and inverse of frequency (rows). Parameters for cepstrum calculation were as follows: 1024-sample Slepian window (Matlab function dpss with number of windows = 1), 984-sample overlap between windows, 1024 frequency points for the first Fourier transform, and 8192 points for the second Fourier transform. For each column of the cepstrum matrix, we then identified the maximum value between 400 and 3000 Hz^−1^ and defined the fundamental frequency as the inverse of the position of the maximum. We also defined the goodness of pitch [46] as the fraction of the total sound power between 400 and 3000 Hz that was carried by harmonics of the fundamental frequency; for an ideal harmonic stack, the goodness of pitch is 1. The parameters we used resulted in 1-ms time resolution for traces of fundamental frequency and for traces of the goodness of pitch. To improve alignment of the measurements across different motif renditions, all traces were linearly time warped to the same number of samples. Harmonic stacks were then defined as periods of time that satisfied the following criteria: 1) At each time point in the stack, at least 90% of all recorded motifs had goodness of pitch values exceeding 0.5. 2) For the difference between neighboring values of fundamental frequency, the median across motifs was between -0.25 and 0.25 Hz/ms at all time points (i.e., frequency was stable). 3) Conditions 1 and 2 were satisfied for a continuous period of at least 20 ms. For syllables that were broken into multiple harmonic stacks, we only used the longest stack for analysis.

As was done in previous studies [5], [8], [13], we quantified acoustic variability of a given set of harmonic stacks by calculating the coefficient of variation (CV, defined as standard deviation/mean) of all fundamental frequency values.

## Supporting Information

Figure S1 **Relationship between motif duration and temperature across all birds.** Plots show temperature and duration of all directed (red symbols) and undirected (blue symbols) motifs for each of the 8 recorded birds. HVC temperature was recorded in 5 of these birds (green titles; birds #1, #2, #3, #4 and #7). Temperature in the hyperpallium outside of the song system was recorded in the other 3 birds (orange titles; birds #5, #6 and #8). Individual examples presented in other figures are from bird #2 (Figure 3), bird #3 (Figure 2), bird #5 (Figures 2 and 5) and bird #7 (Figure 4).
(TIF)
