# Supplementary figures and images for: Natural Changes in Brain Temperature Underlie Variations in Song Tempo during a Mating Behavior
Source: PLoS One. 2012 Oct 24;7(10):e47856. doi: 10.1371/journal.pone.0047856 (PMC3480430; doi:10.1371/journal.pone.0047856)

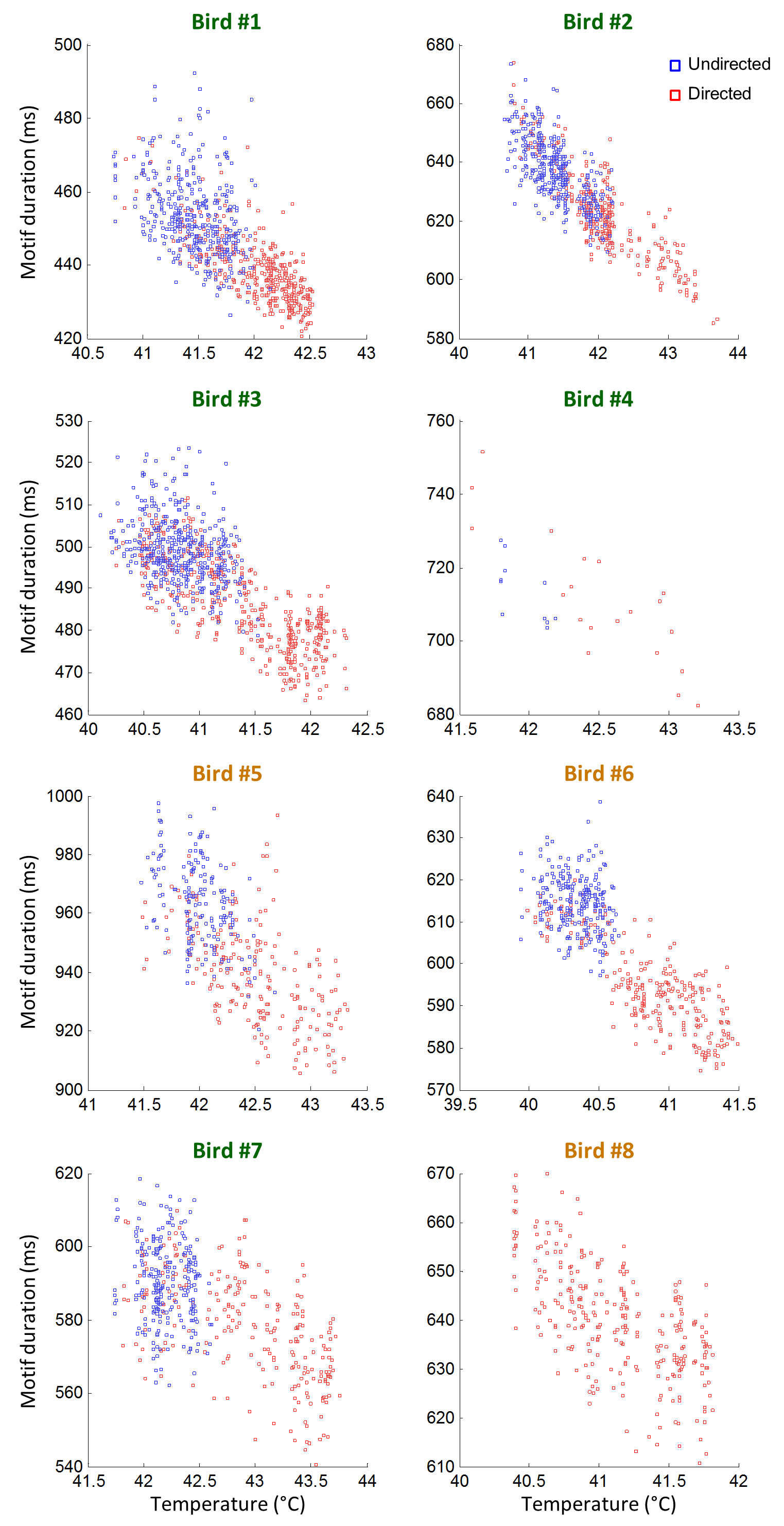

Supplement: Figure S1 — Relationship between motif duration and temperature across all birds. Plots show temperature and duration of all directed (red symbols) and undirected (blue symbols) motifs for each of the 8 recorded birds. HVC temperature was recorded in 5 of these birds (green titles; birds #1, #2, #3, #4 and #7). Temperature in the hyperpallium outside of the song system was recorded in the other 3 birds (orange titles; birds #5, #6 and #8). Individual examples presented in other figures are from bird #2 (Figure 3), bird #3 (Figure 2), bird #5 (Figures 2 and 5) and bird #7 (Figure 4). (TIF) [file pone.0047856.s001.tif]
